# Mapping Pectic-Polysaccharide Epitopes in Cell Walls of Forage Chicory (*Cichorium intybus*) Leaves

**DOI:** 10.3389/fpls.2021.762121

**Published:** 2021-11-22

**Authors:** Xuezhao Sun, Ian G. Andrew, Philip J. Harris, Simone O. Hoskin, Keith N. Joblin, Yuhua He

**Affiliations:** ^1^The Innovation Centre of Ruminant Precision Nutrition and Smart and Ecological Farming, Jilin Agricultural Science and Technology University, Jilin City, China; ^2^Jilin Inter-Regional Cooperation Centre for the Scientific and Technological Innovation of Ruminant Precision Nutrition and Smart and Ecological Farming, Jilin City, China; ^3^Retired Researcher, Palmerston North, New Zealand; ^4^School of Biological Sciences, The University of Auckland, Auckland, New Zealand; ^5^Independent Researcher, Palmerston North, New Zealand; ^6^Grasslands Research Centre, AgResearch Limited, Palmerston North, New Zealand

**Keywords:** arabinan, chicory, galactan, homogalacturonan, immunolabelling, pectins

## Abstract

The cell walls of forage chicory (*Cichorium intybus*) leaves are known to contain high proportions of pectic polysaccharides. However, little is known about the distribution of pectic polysaacharides among walls of different cell types/tissues and within walls. In this study, immunolabelling with four monoclonal antibodies was used to map the distribution of pectic polysaccharides in the cell walls of the laminae and midribs of these leaves. The antibodies JIM5 and JIM7 are specific for partially methyl-esterified homogalacturonans; LM5 and LM6 are specific for (1→4)-β-galactan and (1→5)-α-arabinan side chains, respectively, of rhamnogalacturonan I. All four antibodies labelled the walls of the epidermal cells with different intensities. JIM5 and JIM7, but not LM5 or LM6, labelled the middle lamella, tricellular junctions, and the corners of intercellular spaces of ground, xylem and phloem parenchyma. LM5, but not LM6, strongly labelled the walls of the few sclerenchyma fibres in the phloem of the midrib and lamina vascular bundles. The LM5 epitope was absent from some phloem parenchyma cells. LM6, but not LM5, strongly labelled the walls of the stomatal guard cells. The differential distribution of pectic epitopes among walls of different cell types and within walls may reflect the deposition and modification of these polysaccharides which are involved in cell wall properties and cell development.

## Introduction

The cell walls of flowering plants contain polysaccharides together with other substances such as phenolic components, proteins, and glycoproteins. The polysaccharides comprise cellulose, and non-cellulosic polysaccharides, including pectic polysaccharides, and hemicelluloses. The non-cellulosic polysaccharides are usually very different in non-lignifed primary walls and lignified secondary walls and also vary with plant species. In the primary cell walls of eudicotyledons and non-commelinid monocotyledons, pectic polysaccharides are major components (Harris, 2005).

Pectic polysaccharides have a domain structure consisting of homogalacturonan (HG), rhamnogalacturonan-I (RG-I), rhamnogalacturonan-II (RG-II), and sometimes small amounts of xylogalacturonan and apiogalacturonan (Caffall and Mohnen, 2009). HG is primarily a (1→4)-α-linked polymer of galacturonic acid residues, which may be methyl esterified (Hocq et al., 2017). When synthesised in the Golgi apparatus, HG is highly methyl-esterified and is at least partially de-esterified in the cell wall by the actions of pectin methylesterases. The degree of methyl esterification has important implications in regard to location and function in the walls (Levesque-Tremblay et al., 2015). RG-I is a branched polymer with a backbone of alternating galacturonic acid and rhamnose residues, the latter bearing (1→5)-α-arabinan and/or (1→4)-β-galactan side-chains (Silva et al., 2016). RG-II is a complex polymer involved in linking chains by way of borate ester bridges (O’Neill et al., 2004; Mohnen, 2008). Specific pectic polysaccharides can be localised within cell walls using monoclonal antibodies that recognise specific structures (epitopes) within them. The monoclonal antibodies JIM5 and JIM7 recognise a range of partially methyl-esterified HG structures (Willats et al., 2000; Clausen et al., 2003). JIM5 binds optimally to at least four contiguous (1→4)-α-D-galacturonic acid (GalA) residues that are not esterified between or adjacent to a methyl-esterified GalA residue; it also binds weakly to unesterified HG. However, JIM7 binds to structures where every second GalA residue is methyl esterified and there is no preference for the esterification state of the GalA residue between (Clausen et al., 2003). The monoclonal antibodies LM5 and LM6 bind to the (1→4)-β-galactan and (1→5)-α-arabinan side-chains of RG-I, respectively. LM5 binds to an epitope with a minimum of three residues at the non-reducing end of a linear (1→4)-β-galactan (Jones et al., 1997; Andersen et al., 2016), and LM6 binds to an epitope with about five residues within a linear (1→5)-α-arabinan (Willats et al., 1998).

Forage chicory (*Cichorium intybus*), a plant in the family Asteraceae, is an emerging crop used as a forage herb (Li and Kemp, 2005). It is degraded fast in the rumen (Sun et al., 2008) and consequently has a high feed intake by ruminants, resulting in a high feeding value (Barry, 1998). Our previous studies on the chemical composition of chicory leaf cell walls showed that pectic polysaccharides account for 67% of the total wall polysaccharides in the laminae and 58% in the midribs (Sun et al., 2006). A methylation analysis suggested these pectic polysaccharides are heterogeneous, with mainly HG, and RG-I with sidechains of (1→4)-β-galactans and (1→5)-α-arabinans. Further studies on chicory leaves treated with endopolygalacturonanase (Sun et al., 2007) and rumen bacteria (Sun et al., 2008) suggested that the rapid breakdown of chicory leaves in the rumen is probably because of the high content of pectic polysaccharides in their cell walls and also possibly the distribution of these polysaccharides in the cell walls (Li, 2021). However, little is known about the distribution of pectic polysaccharides in chicory leaf tissues and cell walls. Understanding this distribution is essential for clarification of their biological functions (Knox, 2008) and their degradation by rumen bacteria. In this study, we used immunolabelling to map HG epitopes having different degrees of esterification with JIM5 and JIM7, and epitopes of galactan and arabinan side-chains of RG-I with LM5 and LM6.

## Materials and Methods

### Plant Material

Forage chicory (*C. intybus* L. cv. Puna II) seed was sown in trays (30 cm × 40 cm, 10 cm deep) containing potting mix, which was placed in an unheated, naturally-lit glasshouse for 4 weeks and then outside for a further 4 weeks. The youngest, fully expanded leaves were harvested from five plants.

### Monoclonal Antibodies

The monoclonal antibodies JIM5 and JIM7 were kindly provided by Professor K Roberts, John Innes Centre, Norwich, United Kingdom, and LM5 and LM6 by Professor JP Knox, Leeds University, United Kingdom.

### Sections

Transverse segments were cut midway along the leaf midrib, and in the lamina, halfway between the midrib, and the edge of the leaf. The three segments from each plant were fixed and embedded, as described by Trethewey and Harris (2002). Briefly, the samples were cut and fixed in a freshly-prepared fixative containing 2% (w/v) paraformaldehyde and 1% (w/v) glutaraldehyde in 100 mM sodium hydroxide-piperazine-1,4-bis (2-ethanesulfonic acid) (NaOH-PIPES) buffer (pH 7.2) for 4 h, followed by five washes in the buffer solution without fixatives, and then postfixed with or without 1% (w/v) osmium tetroxide in 50 mM NaOH-PIPES buffer (pH 7.2) for 1 h. The segments were washed five times in the same buffer solution, but without the fixatives, and dehydrated using a graded ethanol series (30, 60, 90, 95, and 100%), leaving them for 10 min in each solution. Prior to infiltration, the segments were left in 100% ethanol for 1 h to dehydrate further. The segments were infiltrated with LR White Resin (London Resin Ltd, Basingstoke, United Kingdom) using first a mixture of ethanol and resin (1:1 v/v, for 16 h) and then three changes (12 h each) of pure resin. Mixing was done by rotating the containers. Finally, the segments were embedded using fresh resin in a pre-dried gelatin capsule at 60°C for 2 days.

Semithin sections (0.5 μm thick) of leaf embedded in resin were cut with a diamond knife using an ultramicrotome (Model Ultracut E, Reichert-Jung, Vienna, Austria), collected on a slide coated with polylysine, dried at 60°C, and used for immunofluorescence labelling. Ultrathin sections (70–100 nm thick) for electron microscopy were cut with a diamond knife using an ultramicrotome and collected on Formvar/carbon-coated or uncoated nickel grids (150 mesh).

### Leaf Anatomy

Semithin sections were stained with toluidine blue O [0.05% (w/v), in 20 mM sodium benzoate buffer, pH 4.4] for leaf anatomy (Feder and O’Brien, 1968; O’Brien and McCully, 1981).

### Immunofluorescence Labelling

Semithin sections were washed in 10 mM phosphate-buffered saline (PBS) buffer (pH 7.2, 8 mM Na_2_HPO_4_, 2 mM KH_2_PO_4_, and 150 mM NaCl) for 2 min. Non-specific binding sites were blocked by incubating the sections with 1% (w/v) bovine serum albumin (BSA; Fraction V, Sigma, St Louis, MO, United States) in PBS buffer for 20 min. After washing three times with PBS, the sections were incubated with primary antibodies. JIM5, JIM7, LM5, and LM6 were diluted 10-, 10-, 4-, and 4-fold, respectively, in 1% BSA/PBS before use. Sections were incubated for 2 h at 20°C with JIM5, JIM7, and LM5, and for 16 h at 4°C with LM6, in a moist chamber. The sections were washed again with three changes of PBS (each change 1 min) and incubated first with secondary antibody, biotinylated anti-rat IgG (Amersham International plc, Little Chalfont, United Kingdom), for 1 h, and then with streptavidin fluorescein (Amersham Life Science, Little Chalfont, United Kingdom) for 30 min. Both anti-rat IgG and streptavidin fluorescein were used at dilutions of 1/200 in 1% BSA/PBS. After washing in PBS three times and water once, the sections were mounted in Vectashield mounting medium for fluorescence microscopy (Vector Laboratories, Burlingame, CA, United States) and examined with a conventional fluorescence microscope (wide-field fluorescence microscope; Model BX51; Olympus, Shinjuku-ku, Tokyo, Japan) fitted with a BP 460–490 nm excitation filter, a DM 505 nm chromatic beam splitter, and a BA 510–550 nm barrier filter. Photomicrographs were taken with a digital microscope camera (Optronics; Olympus U-TV.5XC). Controls were done in which incubation with the primary antibody, or the secondary antibody was omitted.

### Immunogold Labelling

The ultrathin sections were washed with PBS-T buffer [pH 7.4, 16 mM Na_2_HPO_4_, 4 mM KH_2_PO_4_, 0.1% (w/v) BSA, 0.1% (v/v) Tween 20, 150 mM NaCl, and 15 mM NaN_3_, passed through a filter (pore size 0.22 μm)], then preincubated for 30 min at room temperature with PBS-T buffer containing 1% (w/v) BSA (BSA/PBS-T) to block non-specific binding sites. The sections were then incubated at 4°C overnight with the primary antibodies [dilution 1:10, 1:10, 1:4, and 1:4 (v/v) for JIM5, JIM7, LM5, and LM6, respectively]. After washing with PBS-T (five times, 2 min for each wash), the sections were incubated for 2 h at 20°C with goat anti-rat IgG (H&L) conjugated to 1 nm colloidal gold (Electron Microscopy Sciences, Fort Washington, PA, United States) at 1:50 dilution in BSA/PBS-T. The sections were then washed with PBS-T and water (five times with PBS-T, five times with water, 2 min for each wash). After drying, the sections were silver enhanced with a silver enhancement kit (BB International Ltd., United Kingdom) for 3 min in the dark at 20°C, followed by washing with water (three times) in darkness and then whilst holding with shaking forceps for 1 min. After drying, the sections were examined using a transmission electron microscope (Philips TEM 201C, Eindhoven, Netherlands) at an operating voltage of 60 kV. Controls were done by either omitting the incubation with the primary monoclonal antibody or by omitting the incubation with the secondary antibody.

## Results

**FIGURE 1:**
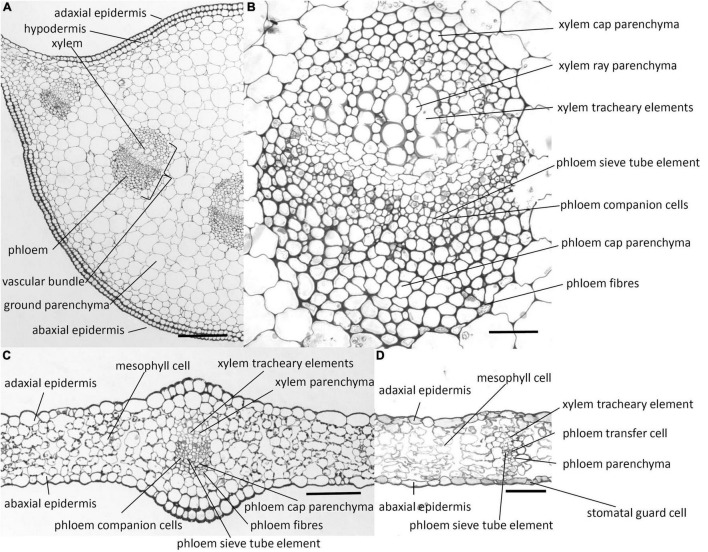
Photomicrographs of transverse sections cut from a rosette leaf of an 8-week-old chicory plant. **(A)** A transverse section of the midrib. **(B)** A vascular bundle in the midrib. **(C)** A transverse section of the lamina with a major vein. **(D)** A transverse section of the lamina with a minor vein. Although it is difficult to identify the transfer cells, sieve tube elements, and companion cells in the photomicrographs, the lines point to where these cell types occur. Bars: **(A)** 200 μm; **(B)** 40 μm; and **(C,D)** 100 μm.

Transverse sections of the midrib and lamina of the chicory leaf are shown in Figure 1. In the midrib (Figure 1A), there is a hypodermal layer beneath the epidermis. Between the ab- and adaxial hypodermal layers, there are vascular bundles surrounded by ground parenchyma cells. Both the phloem and xylem tissues of the vascular bundles have caps composed of parenchyma cells with walls that are thicker than those of the ground parenchyma (Figure 1B). These cap parenchyma cells resemble collenchyma cells and have been referred to as collenchymatous (Esau, 1965). Phloem fibres occur as a broken crescent surrounding the phloem cap. These fibres have thick cell walls which are not lignified as indicated by the toluidine blue staining and the phloroglucinol-HCl colour reaction (see below). The phloem comprises sieve tube elements and companion cells. The xylem comprises tracheary elements and parenchyma, which occurs between the tracheary elements. The walls of the xylem tracheary elements were the only walls that gave a histochemical reaction for lignin using the colour reagent phloroglucinol-HCl (Sun et al., 2006).

In contrast to the midrib, the lamina has no hypodermal layer (Figures 1C,D). Stomatal guard cells are present mostly in the abaxial epidermis, but occasionally in the adaxial epidermis. The lamina also contains vascular bundles, which are smaller than those in the midrib and have fewer cells. The cell types are similar to those in the midrib, but the broken crescent of phloem fibre cells is commonly replaced by solitary less differentiated phloem fibre cells, and the phloem companion cells are transfer cells, with wall ingrowths although the major veins have no transfer cells.

**FIGURE 2:**
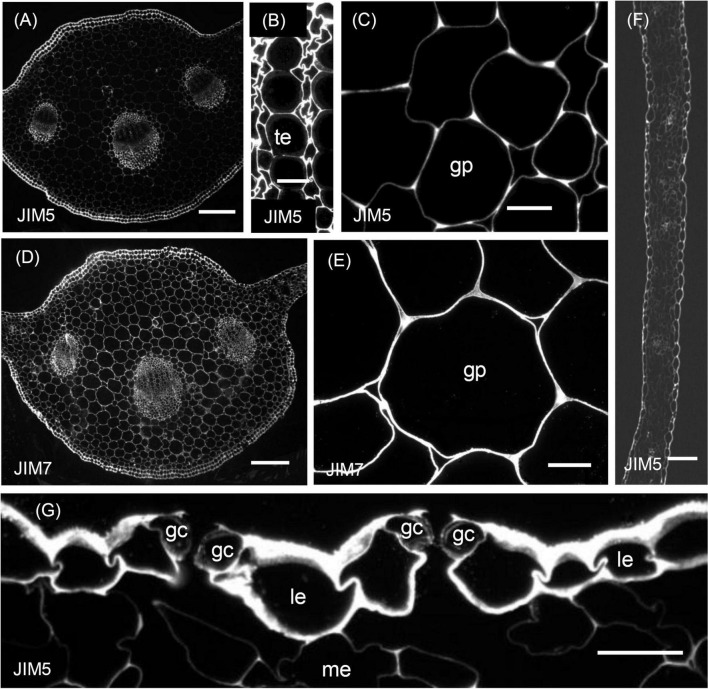
Immunofluorescence micrographs of transverse sections cut from a rosette leaf from an 8-week-old chicory plant and labelled with JIM5 or JIM7. **(A)** The midrib labelled with JIM5. **(B)** Xylem tracheary elements and adjacent xylem parenchyma cells in a vascular bundle in the midrib labelled with JIM5. **(C)** Parenchyma cells (ground parenchyma) between the midrib hypodermis and the vascular bundles, showing more intense JIM5 labelling at the cell corners. **(D)** The midrib labelled with JIM7. **(E)** Parenchyma cells (ground parenchyma cells) between the midrib hypodermis and the vascular bundles labelled with JIM7. **(F)** The lamina, showing moderate labelling of the outer epidermal walls and the walls of cells in the minor veins. **(G)** Stomatal guard cells, showing weak labelling of the walls of stomatal guard cells, with the outermost regions of these walls and outer ledge more intensely labelled. gp: ground parenchyma; gc: stomatal guard cells; le: lamina epidermis; me: mesophyll; and te: tracheary element. Bars: **(A,D)** 200 μm; **(B,C,E,G)** 100 μm.

**FIGURE 3:**
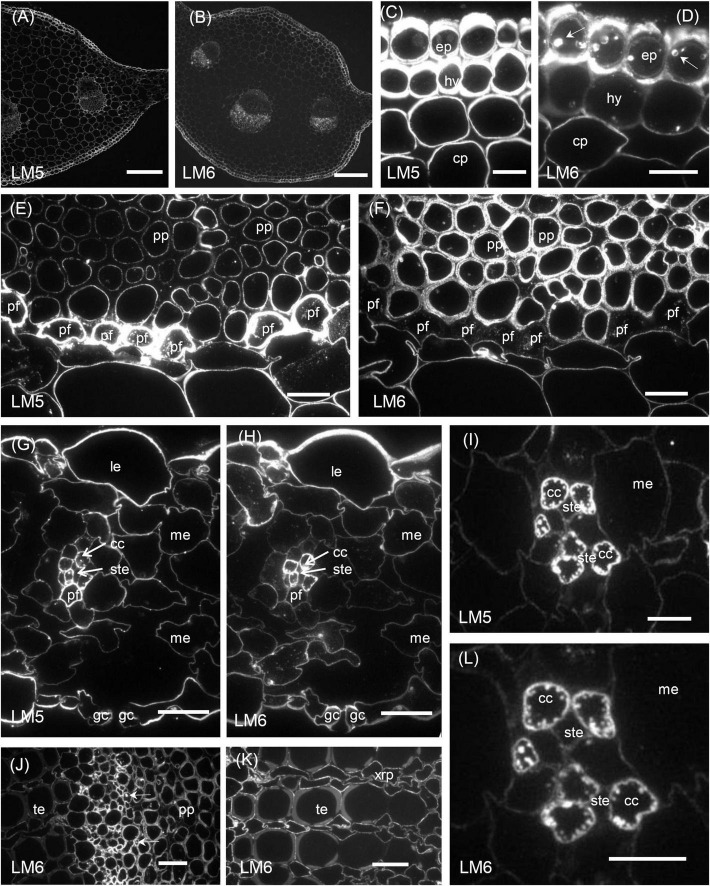
Immunofluorescence micrographs of transverse sections cut from a rosette leaf of an 8-week-old chicory plant and labelled with LM5 or LM6. **(A)** The midrib labelled withLM5. **(B)** The midrib labelled with LM6. **(C)** The midrib labelled with LM5, showing very strong labelling on the epidermal and hypodermal walls and moderate labelling of the parenchyma cell walls. **(D)** The midrib labelled with LM6, showing the very strong labelling of the epidermal walls and the moderate labelling of the hypodermal and parenchymal cell walls. **(E)** The midrib labelled with LM5, showing very strong labelling of the phloem fibre walls and the inner walls of the phloem parenchyma cells. **(F)** The midrib labelled with LM6, showing strong labelling of the phloem parenchyma cell walls but not the middle lamellae between these cells and no labelling of the phloem fibre walls. **(G)** The lamina labelled with LM5, showing strong labelling of the epidermal cell walls and no labelling on the walls of the stomatal guard cells. **(H)** The lamina labelled with LM6, showing intense labelling of the outer walls of the epidermal cells and the walls of the phloem transfer cells in a minor vein. **(I)** A minor vein in the lamina labelled with LM5, showing strong labelling of the walls and wall ingrowths of phloem companion cells (transfer cells). **(J)** The phloem in a midrib vascular bundle labelled with LM6, showing labelling of inclusions in some inner phloem cells. **(K)** The xylem in a midrib vascular bundle labelled with LM6, showing no labelling of the secondary walls of the tracheary elements but labelling of the primary walls of adjacent elements, and the walls of xylem ray parenchyma cells. **(L)** A minor vein in a lamina labelled with LM6, showing strong labelling of the walls and wall ingrowths of phloem transfer cells. cc: phloem transfer cell; ep: epidermal cell; gc: stomatal guard cell; hy: hypodermis; le: lamina epidermis; me: mesophyll; pf: phloem fibre; pp: phloem parenchyma; ste: sieve tube element; te: tracheary element; and xrp: xylem ray parenchyma. Bars: **(A,B)** 200 μm; **(C–H,J,K)** 20 μm; and **(I,L)** 5 μm.

**FIGURE 4:**
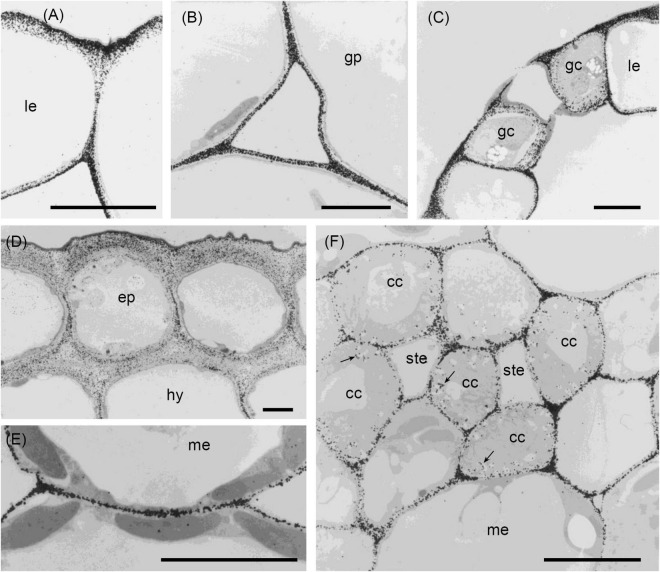
Transmission electron micrographs of transverse sections cut from a rosette leaf of an 8-week-old chicory plant and immunogold labelled with JIM5. **(A)** A lamina epidermal cell, showing labelling in the outer wall increasing toward the outer surface. **(B)** The ground parenchyma in the midrib, showing labelling particularly concentrated at the cell corners and along walls near the middle lamella. **(C)** Stomatal guard cells, showing strong labelling of the outermost regions of the cell walls and throughout the outer ledge and no labelling of the cuticle. **(D)** Epidermis and hypodermis, showing heavy labelling of the middle region of walls between two epidermal cells and between two hypodermal cells. **(E)** Mesophyll cells, showing labelling of the walls that is particularly strong in regions where adjacent cells adhere. **(F)** A minor vein in a lamina, showing labelling of the wall ingrowths of transfer cells. cc: transfer cell; ep: epidermal cell; gc: stomatal guard cell; gp: ground parenchyma; hy: hypodermis; le: lamina epidermis; me: mesophyll; ste: phloem sieve tube element; and →: wall ingrowth. Bars: 5 μm.

**FIGURE 5:**
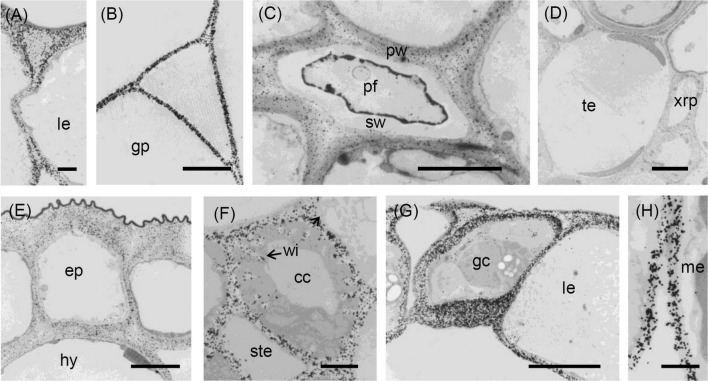
Transmission electron micrographs of transverse sections cut from a rosette leaf of an 8-week-old chicory plant and immunogold labelled with JIM7. **(A)** A lamina epidermal cell, showing the walls evenly labelled. **(B)** A ground parenchyma cell in the midrib, showing labelling throughout the walls but less intense at the corners of cell junctions. **(C)** A phloem fibre, showing labelling in the primary walls but no labelling in the secondary walls. **(D)** The xylem in the midrib, showing labelling in the primary walls of the tracheary elements and xylem ray parenchyma cells but no labelling in the secondary walls of the tracheary elements. **(E)** Epidermal and hypodermal cells, showing even labelling of the walls. **(F)** Transfer cells in a minor vein of the lamina, showing labelling of transfer cell walls and wall ingrowths. **(G)** Stomatal guard cells, showing dense, but non-uniform labelling of the guard cell walls, with labelling also present in the cuticular layer. **(H)** Mesophyll cells, showing no labelling at the corners of intercellular spaces. cc: transfer cell; gp: ground parenchyma; ep: epidermal cell; gc: stomatal guard cell; hy: hypodermis; le: lamina epidermis; me: mesophyll; pf: phloem fibre; pw: primary wall; sw: secondary wall; te: tracheary element; xrp: xylem ray parenchyma; and wi: wall ingrowth. Bars: **(A,F,H)** 1 μm; **(B–E,G)** 5 μm.

**FIGURE 6:**
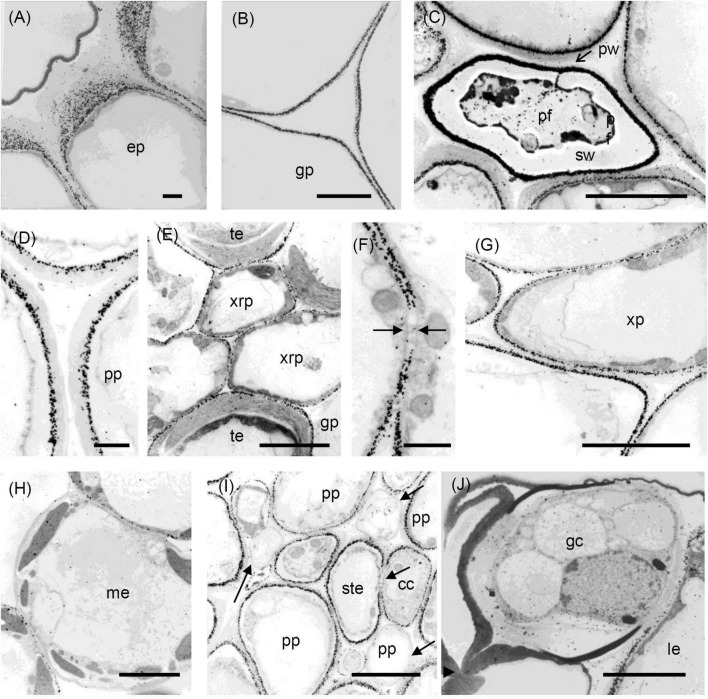
Transmission electron micrographs of transverse sections cut from a rosette leaf of an 8-week-old chicory plant and immunogold labelled with LM5. **(A)** The outer wall of epidermal cells in the midrib, showing that labelling gradually increased from the cuticle to the plasma membrane, but was largely absent from the middle lamella. **(B)** A ground parenchyma cell in the midrib, showing labelling of the primary walls, but no labelling of the middle lamella or the corners of the intercellular spaces. **(C)** A phloem fibre, showing that labelling was heavy and in a narrow region of the primary walls. **(D)** A parenchyma cell in the phloem cap, showing thick walls with labelling confined to the region near the plasma membrane. **(E)** The xylem, showing that labelling is absent in the secondary walls of the tracheary elements, but present in the primary walls of tracheary elements and xylem ray parenchyma cells. **(F)** A ground parenchyma cell, showing no labelling of the pit field. **(G)** A xylem parenchyma cell, showing the same pattern of labelling as in **(B)**. **(H)** Mesophyll cells, showing that most walls were labelled, but a few were not. **(I)** Phloem parenchyma cells near the sieve tube elements, showing no labelling of the walls of some of the phloem parenchyma cells. **(J)** Stomatal guard cells, showing no labelling of the walls. cc: transfer cell; gp: ground parenchyma; ep: epidermal cell; gc: stomatal guard cell; le: lamina epidermis; me: mesophyll cell; pf: phloem fibre; pp: phloem parenchyma; pw: the primary wall; ste: sieve tube element; sw: the secondary wall; te: tracheary element; xrp: xylem ray parenchyma; and xp: xylem parenchyma; arrows show the absence of labelling. Bars: **(A,D,F)** 1 μm; **(B,C,E,G–J)** 5 μm.

**FIGURE 7:**
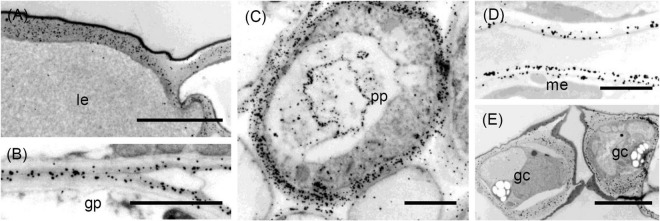
Transmission electron micrographs of transverse sections cut from a rosette leaf of an 8-week-old chicory plant and immunogold labelled with LM6. **(A)** An epidermal cell in the lamina, showing dense labelling of the outer walls, but light labelling at the junction of two cells. **(B)** Ground parenchyma cells, showing labelling present in the outer region of the primary walls but no labelling of the corners of intercellular spaces. **(C)** A phloem parenchyma cell, showing heavy labelling of the walls **(D)** Mesophyll cells in the lamina, showing labelling in the walls; this labelling appears as two lines in some walls. **(E)** Stomatal guard cells, showing heavy labelling of the walls. gp: ground parenchyma; gc: stomatal guard cell; le: lamina epidermis; me: mesophyll; and pp: phloem parenchyma. Bars: **(A,E)**: 5 μm; **(B–D)**: 2 μm.

The results of the immunofluorescence labelling are shown in Figures 2, 3 and the results of the immunogold labelling in Figures 4, 5, 6, 7. Table 1 summarises these results. No immunofluorescence or immunogold labelling was observed in control sections when either the primary antibody or the secondary antibody was omitted.

**TABLE 1:** Immunofluorescence and immunogold labelling with JIM5, JIM7, LM5, and LM6 of the cell walls in transverse sections cut from rosette leaves of an 8-week-old chicory plant.

		Monoclonal antibody			
Region of leaf	Cell type	JIM5	JIM7	LM5	LM6
Midrib	Epidermis (adaxial and abaxial)	++++	++++	++++	++++
	Hypodermis	++++	++++	++++	++
	Ground parenchyma (cell corners)	++ (+++)	+++ (++/+++)	++ (–)	++ (+)
	Vascular bundles				
	Phloem cap parenchyma	+++	+++	++^a^	++
	Phloem fibres (outer region of cap, primary walls)	+++	+++	++++	–
	Phloem fibres (outer region of cap, secondary walls)	–	–	–	–
	Phloem sieve tube elements	+++	+++	++	+++
	Phloem companion cells and other phloem parenchyma	+++	+++	++	+++
	Xylem cap parenchyma	+++	+++	++	++
	Xylem parenchyma between xylem tracheary elements	+++	+++	++	++
	Xylem tracheary elements (primary walls)	++	++	++	++
	Xylem tracheary elements (secondary walls)	–	–	–	–
Lamina	Epidermis (adaxial and abaxial)	+++	++	++	+++
	Stomatal guard cells	+	++	–	+++
	Mesophyll cells	+	+	+	+
	Vascular bundles				
	Phloem cap parenchyma	+	+	+	+
	Phloem fibres (outer region of cap, primary walls)	+++	+++	++++	–
	Phloem fibres (outer region of cap, secondary walls)	–	–	–	–
	Phloem sieve tube elements	++	+++	+	+
	Phloem companion cells (transfer cells in minor veins)	++	+++	+++	+++
	Xylem parenchyma	+	+	+	+
	Xylem tracheary elements (primary walls)	+	+	+	+
	Xylem tracheary elements (secondary walls)	–	–	–	–

*Labelling intensity: ++++, very strong; +++, strong; ++, moderate; +, weak; and –, absent.*

*^a^Labelling confined to wall region adjacent to the plasma membrane.*

### Distribution of the JIM5 Epitope

In sections of the midrib, immunofluorescence labelling of the walls of the epidermal and hypodermal cells was very strong (Table 1 and Figure 2A), but the labelling of the walls of the ground parenchyma cells was only very strong at the cell corners (Figure 2C). In the vascular bundles, the walls of the xylem and phloem cap parenchyma cells and the walls of the xylem parenchyma cells between the tracheary elements were strongly labelled (Figure 2A). In the phloem fibres and xylem tracheary elements, which had both primary and secondary walls, only the primary walls were labelled (Figure 2B). In sections of the lamina, labelling was greatest in the epidermal walls and, in the major veins, in the primary walls of the phloem fibres (Figure 2F). However, the stomatal guard cell walls were only weakly labelled (Figure 2G).

Immunogold labelling showed that in all cell types, the labelling was most intense in the middle lamella (Figure 4) and cell walls adjacent to intercellular spaces (Figure 4B). The labelling was often more intense at the corners of the intercellular spaces (Figure 4B). The middle lamella region of the radial walls of the epidermis and hypodermis was also labelled conspicuously, but the labelling of the contiguous tangential walls of the epidermis and hypodermis was more diffuse, spreading unevenly throughout the walls (Figure 4D). The labelling was less in the middle lamella region and more intense in the region closer to the cytoplasm in the tangential walls. Labelling was particularly intense throughout the outer walls of the epidermis, with a definite narrow zone of higher intensity immediately adjacent to the cuticle (Figure 4D). In the lamina, the JIM5 epitope was present in all mesophyll cell walls, where it was concentrated in the middle lamella and at all the corners (Figure 4E). Labelling was most intense in the outer walls of the epidermis (Figure 4C), especially in the outer half (Figure 4A) and where the wall thickened next to the radial walls. In stomatal guard cell walls (Figure 4C), labelling was intense in the outermost regions of the cell walls and throughout the outer ledge but absent from the cuticle. In minor veins of the lamina, labelling was most intense at the tricellular junctions among phloem sieve tube elements and companion cells (transfer cells) but labelling extended throughout the middle lamella region of phloem sieve tube elements and transfer cell walls, as well as the wall ingrowths of the latter (Figure 4F).

### Distribution of the JIM7 Epitope

Immunofluorescence labelling with JIM7 was similar to that with JIM5, except the walls of the ground parenchyma cells in the midrib were more strongly labelled. In the lamina, labelling of the epidermal walls was less than with JIM5, but the labelling of the stomatal guard cell walls was greater. Unlike JIM5, immunogold labelling of the middle lamella was not more intense than the primary walls (Figure 5B). The immunofluorescence labelling with JIM7 showed the epitope was present in the primary walls of all cell types and usually uniformly distributed throughout the primary wall (Figure 2D), but not the middle lamella. Both immunofluorescence (Figure 2E) and immunogold (Figure 5B) labelling show less labelling in the middle of the wall region between cells at the corners, contrasting strongly with JIM5, which showed more intense labelling in these regions (Figure 2C). The epitope was more uniformly distributed among cell types than the JIM5 epitope. The JIM7 epitope was especially abundant in epidermal cell walls (Figure 2D). In contrast to JIM5, labelling of the walls of the ground parenchyma cells by JIM7 appeared uniform throughout.

Immunogold labelling showed that the epidermal walls were heavily and almost uniformly labelled with JIM7 (Figure 5E), but with greater intensity in the more recently laid down wall regions close to the plasma membrane. In the ground parenchyma cells, the JIM7 epitope was distributed throughout the walls but tended to be concentrated away from the middle lamella and the corners of cell junctions (Figure 5B). A similar pattern was observed in the phloem parenchyma cell walls. The JIM7 epitope was absent from the phloem fibres (Figure 5C) and the secondary walls of xylem vessels (Figure 5D).

In contrast to JIM5, JIM7 labelling was dense and fairly uniformly distributed in lamina epidermal walls (Figure 5A), but in the outer walls, it was less dense opposite the junction of two cells (Figure 5A). Stomatal guard cell walls exhibited a dense, but non-uniform labelling with JIM7, with the epitope present in the cuticular layer (Figure 5G). In phloem cells of the lamina, the JIM7 epitope was distributed throughout the walls of sieve tube elements and transfer cells as well as in the wall ingrowths of the latter (Figure 5F). The JIM7 epitope did not accumulate at the corners of intercellular spaces of mesophyll cells (Figure 5H).

### Distribution of the LM5 Epitope

Immunofluorescence labelling with LM5 was present in the primary walls of most cell types in the midrib (Figure 3A), with the greatest density of labelling in walls of the epidermis, hypodermis (Figure 3C), and phloem fibres (Figure 3E), whereas ground parenchyma cells and vascular bundles were relatively uniformly labelled (Figure 3A). LM5 labelling between cells generally took the form of a double line, with a gap down the middle lamella region (Figure 3C), in contrast to the single line with JIM5. There was no labelling of the corners of intercellular spaces. In the phloem parenchyma, the LM5 labelling was confined to the extreme inner part of the walls (Figure 3E). In the lamina, the LM5 epitope was present in the walls of most cells but most abundant in the outer epidermal walls, and largely absent from the stomatal guard cells (Figure 3G). The epitope was also dense in the walls and wall ingrowths of transfer cells in minor veins of the lamina (Figure 3I).

Immunogold labelling showed the LM5 epitope to concentrate in the regions of walls near the cytoplasm of most cell types, including ground parenchyma cells (Figure 6B), phloem (Figure 6D) and xylem parenchyma cells (Figure 6G) but was absent from the middle lamella region and corners of intercellular spaces (Figures 6B,D,G). In the epidermis this appeared as a gradation of labelling intensity in the outer epidermal walls, increasing from the cuticle to the plasma membrane (Figure 6A). In the ground parenchyma cells, the labelling took the form of two parallel lines, sometimes interrupted by a short zone, apparently corresponding to a pit field (Figure 6F). In the phloem, the fibres showed very dense labelling as a band in their primary walls (Figure 6C), but no labelling in their secondary walls; indeed, none of the four antibodies used labelled these secondary walls. Most inner phloem cell walls, including those of the sieve tube elements and companion cells, showed strong labelling, largely confined to the inner wall, but the walls of some (mostly smaller) inner phloem cells were not labelled (Figure 6I). In the xylem, the LM5 epitope was found in the primary walls of tracheary elements, but not in the secondary walls (Figure 6E). In the lamina, immunogold labelling showed the LM5 epitope was much less dense in the stomatal guard cell walls than in the walls of adjacent epidermal cells (Figure 6J). Mesophyll walls were generally uniformly labelled with LM5, but a few lacked the epitope (Figure 6H).

### Distribution of the LM6 Epitope

In the midrib, immunofluorescence labelling with LM6 was most intense in the walls of the epidermis and phloem cells of the midrib (Figure 3B). It was also moderately intense in the thick walls of parenchyma cells in the outer xylem. Labelling of the ground parenchyma cell walls was more intense in the outer (cortical) regions of the midrib. Inner ground parenchyma cells and xylem parenchyma cells were only weakly labelled. In contrast to LM5, LM6 showed relatively weak labelling of the hypodermal walls (Figure 3D). The fluorescence formed a single line between the ground parenchyma cells and not a double line as with LM5. In the xylem, the LM6 epitope continued along the primary walls between vessels and the walls of xylem ray parenchyma cells (Figure 3K). LM6 labelling was particularly strong in the thick walls of the phloem parenchyma cap cells (Figure 3F) and the thinner walls of the inner phloem parenchyma (Figure 3J). LM6 labelling was also found on inclusions in many inner phloem cells (as indicated with arrows in Figure 3J) and in epidermal cells (Figure 3D). The walls of the LM5 positive phloem fibres did not bind LM6 (Figure 3F, cf. Figure 3E). In the lamina, the most intensely labelled cell walls were the outer epidermal walls and the stomatal guard cell walls, as well as the walls of the phloem transfer cells (Table 1 and Figure 3H), including the wall ingrowths (Figure 3L).

Immunogold labelling showed that the LM6 epitope was evenly distributed in the epidermal walls of the midrib (data not shown). In the ground parenchyma cells, the epitope was present in the middle lamellae, but, unlike the JIM5 epitope, was not specifically concentrated in the corners of intercellular spaces (Figure 7B). In the inner phloem parenchyma cells, some walls were heavily labelled with LM6 (Figure 7C), and many cells also had LM6-positive internal membranes (Figure 7C). In the lamina, the LM6 epitope was found in the outer face of spongy mesophyll walls, but in some cell walls, the second line of epitope was found along the plasma membrane (Figure 7D). In the outer wall of the epidermis, LM6 labelling was dense, except at the junction of two cells (Figure 7A). Dense labelling also occurred in stomatal guard cell walls (Figure 7E). In minor vein phloem, the transfer cell walls, and wall ingrowths were densely labelled, but walls of other cell types were much more sparsely labelled (data not shown).

## Discussion

### Structural Support

In collenchyma and epidermal cells, the thick walls are important in holding the leaf rigid and are possibly strengthened by the pectin hydrogel, with calcium bridging of low methyl HG contributing to wall strength (Markov et al., 2017). A pectin hydrogel can swell and shrink according to ionic conditions and so regulate the porosity and flow of water, which may be important in maintaining turgor pressure (Zwieniecki et al., 2001). Our study showed the JIM5 epitope was more abundant in the walls of the epidermis, hypodermis, and vascular bundles in the midrib and the epidermis of the lamina. It is particularly abundant in the outer walls of the epidermis, and its role may relate to the maintenance of a strong hydrogel and a turgid leaf surface. Similarly, in the midrib of chicory leaves, the high concentration of JIM5 epitope in the thick walls of the parenchyma cells of the phloem cap may assist in holding the cell walls together against turgor pressure, as postulated in celery collenchyma where pectin has been suggested as having a role in controlling the thickness of the cell walls (Jarvis, 1992). The caps of thick-walled parenchyma cells over both the phloem and xylem of vascular bundles in the midrib probably have a role in strengthening the tissues and supporting the leaf.

It has been shown that the CDTA-soluble fraction of peach (*Prunus persica*) fruit walls readily forms gels on treatment with pectin methylesterase, which would be expected to enhance the content of the JIM5 epitope (Zhou et al., 2000). We previously showed that about 50% of the cell walls of chicory leaves were HG and that over half of this was extracted by CDTA, suggesting that much of it was a low methyl polyanionic HG bonded by calcium bridges (Sun et al., 2006). JIM5 would be expected to bind strongly to this. The results reported here suggest a very high content of low methyl HG in the epidermal cell walls and the thick walls of the parenchyma cells of the phloem cap.

In stomatal guard cells, the distribution of the JIM5 epitope, mostly in the outermost part of the cell walls, but not in the cuticle, contrasts with a report from sugar beet (*Beta vulgaris*) leaves, where the JIM5 epitope was found to be restricted to the cuticle of stomatal guard cells (Majewska-Sawka et al., 2002). Our results are consistent with the findings on the eudicotyledon *Vigna sinensis* by Giannoutsou et al. (2020). In our study, the JIM7 epitope was usually dense throughout the stomatal guard cell walls, including the cuticular layer.

### Arabinans and Galactans in Cell Development

We showed that the LM6 epitope (arabinans) was laid down mostly in the oldest regions of the cell walls near the middle lamella, whereas the LM5 epitope (galactans) was only in more recently deposited regions of primary cell walls, near the plasma membrane, and was absent from the middle lamella and intercellular spaces. Although the galactans showed relatively uniform distribution among cell types, a number of cell types were present where either galactans or arabinans were the predominant RG-I epitope. In some cell types, LM5 labelling was undetectable, suggesting the absence of galactans. These included a few mesophyll cells, as well as some small phloem cells. These may have been younger, developing cells, and the galactans had not yet been deposited. However, the galactans were also absent from the primary walls of mature xylem tracheary elements, suggesting a secondary loss.

Other studies have shown that the LM5 epitope was also completely absent in walls newly formed from mesophyll-derived protoplasts of sugar beet (Majewska-Sawka and Münster, 2003). The within cell wall distribution of arabinans compared with galactans found in the present study is consistent with the results of studies on tomato (*Solanum lycopersicum*) fruit (Jones et al., 1997; Wang et al., 2019), potato (*Solanum tuberosum*) tuber tissue (Buffetto et al., 2015), pea (*Pisum sativum*) seeds (Lee et al., 2016), and flax (*Linum usitatissimum*; Vicré et al., 1998). These studies found that the (1→4)-β-galactan was restricted to a thin layer close to the plasma membrane. The pattern of distribution of the LM5 epitope in chicory cell walls also suggested that the galactans were laid down at a particular stage of development, but the localisation to the inner margin of the wall could also be due to the turnover of the galactan. Thus, the thick walls of the parenchyma cells in the phloem cap may be derived from the thin walls of inner phloem cells of younger leaves (Esau, 1969), yet the galactans are still confined just to the extreme inner part of the walls.

In the apex of carrot (*Daucus carota*) roots and suspension cultures, the walls of meristematic or proliferating cells lacked galactans, but contained abundant arabinans, whereas in elongated cells the reverse was the case (Willats et al., 1999). The authors suggested that a developmental switch occurred in the suspension culture when growth hormones were withdrawn, leading to both cell elongation and a change from arabinan to galactan deposition. Our observations of the localisation of arabinans to the outer primary wall regions of most walls in chicory leaf suggest that they were laid down early in development. This is consistent with the finding that the LM6 epitope was particularly prominent in the newly formed walls of sugar beet cells generated from mesophyll-derived protoplasts (Majewska-Sawka and Münster, 2003). The walls of leaf mesophyll and protoplast-derived callus lacked the LM5 epitope and were particularly rich in the LM6 epitope (Majewska-Sawka and Münster, 2003).

It has also been found that galactan is deposited later than arabinan during tuberisation in potatoes (Vincken et al., 2003). They proposed a model in which pectic molecules cannot move freely as they are anchored in cell walls and they suggested this may involve linkage of galactan RG-I side chains to cellulose. Therefore, later deposition of galactans causes the galactans to be highly concentrated near the plasma membrane. In support of this model, we reported that, in chicory cell walls, much of the galactan was tightly associated with cellulose-containing residues after extraction of most of the pectic polysaccharides (Sun et al., 2006). Here we showed that epidermal cell walls have a much broader distribution of the LM5 epitope than those of other cell types. This suggests that the galactan was deposited over a much more extended period in the epidermis than in most cell types. Alternatively, the galactan in the epidermal outer wall may have been more mobile than in other walls allowing it to diffuse partway across the wall before being anchored onto the cellulose.

### Arabinan-Rich and Galactan-Poor Cell Walls in the Phloem, Stomatal Guard Cells, and Pit Fields

The walls of most inner phloem cells of the midrib, including the sieve tube elements, showed both the LM5 and the LM6 epitope, indicating the presence of galactans and arabinans, respectively. However, some inner phloem cells in the midrib lacked the LM5 epitope. In contrast, both the companion cells in the midrib and transfer cells in the minor veins of the lamina showed intense LM6 labelling of both the wall and, in some cases, the cytoplasmic contents.

The companion cells of minor veins in the lamina of chicory, as in other members of the family Asteraceae (Turgeon et al., 2001), are transfer cells, with wall ingrowths to facilitate apoplasmic loading of the sieve tube elements (McCurdy et al., 2008). We observed that these transfer cell walls were strongly labelled with LM6, compared with the much weaker labelling of the walls of mesophyll cells and sieve tube elements, and the labelling extended into the wall ingrowths. The ingrowths appeared to be similar to the peripheral walls in their labelling with JIM5, JIM7, and LM6. This distribution pattern of pectic epitopes is, however, inconsistent with the findings of Vaughn et al. (2007) for the epidermal transfer cells in mature cotyledons of broad bean (*Vicia faba*). These authors found almost no labelling of the wall ingrowths with JIM5 although these were labelled with JIM7, LM5, and LM6.

In the chicory midrib, the thick walls of the parenchyma cells in the phloem caps showed strong labelling with LM6 (arabinans) and weak labelling with LM5 (galactans), with the LM5 epitope confined to the extreme inner edges of the walls. Thus, if these cells were derived from thin-walled inner phloem parenchyma cells in the developing leaf, there must have been a turnover of galactan or redistribution in the wall.

In active shoots of aspen (*Populus tremula* × *P. tremuloides*), it has been found that arabinans became “prevalent at a very early stage in cells committed to differentiate into phloem” and were particularly abundant in walls of sieve tube elements, but galactans were absent (Ermel et al., 2000). These observations suggest that the presence of (1→5)-α-arabinans in the cell walls may be related to phloem cell differentiation. Although this could also be true in chicory, our findings are rather different in that the transfer cell walls in chicory leaves were much more heavily labelled with LM6 than were the walls of the sieve tube elements. Oomen (2003) found the LM5 epitope in the walls of phloem sieve tube elements in potato stolons but not in potato tubers.

Another cell type in chicory leaves with walls that labelled strongly with LM6 (arabinans) but weakly with LM5 (galactans) was the stomatal guard cell. This finding is consistent with a study by Giannoutsou et al. (2016) who found that LM6 labelled stomatal guard cells in maize (*Zea mays*). Stomatal guard cells of chicory also had cytoplasmic LM6 positive inclusions, similar to those in the inner phloem, and the JIM7 and LM6 wall epitopes were markedly resistant to extraction by sodium carbonate (unpublished data). Stomatal guard cell walls need to be particularly flexible since they undergo stresses and reversible changes during stomatal opening and closing (Rui et al., 2018). Degradation of cell-wall arabinans prevented either stomatal opening or closing. It has further been suggested that arabinan side chains of RG-I separate two neighbouring stretches of HG to impede the formation of tight associations, which results in the flexibility of these walls (Jones et al., 2003, 2005).

The high arabinan content in the walls of parenchyma cells of the phloem cap in the midrib of chicory leaves, as well as the walls of transfer cells, stomatal guard cells, and meristematic cells, suggest that this polysaccharide may have several distinct functions in cell walls. Its role in phloem cap parenchyma cells is unlikely to be one of maintaining flexibility as postulated for stomatal guard cells, as these thick-walled cells are probably important in maintaining leaf rigidity.

Cytoplasmic arabinans may represent pools in transit or storage, or they could be associated with arabinogalactan protein (AGP) molecules. In a few chicory lamina mesophyll cells, the LM6 epitope was observed along the plasma membrane as well as in the outer epidermal walls, and this could represent (1→5)-α-arabinan side-chains on an AGP (Pereira et al., 2015).

LM5 labelling was also completely absent in some parts of the ground parenchyma cell walls, which appeared to be pit fields (crossed by plasmodesmata). In the pit field in the pericarp walls of the tomato, labelling with LM6 has been found at the inner face of the cells surrounding the pit fields, whereas LM5 labelling was absent (Orfila and Knox, 2000). Such cell type-dependent pectic polysaccharide distribution may, at least in part, be related to the activities of relevant hydrolytic enzymes. For example, a study of the expression of the gene *ARAF1*, which encodes a bifunctional α-L-arabinofuranosidase/β-D-xylosidase (ARAF1) showed it was expressed in certain cell types in the stems and leaves of *Arabidopsis* (Chávez Montes et al., 2008). Furthermore, *araf1* T-DNA insertional mutants showed increased binding of LM6 in the cell walls of these cell types, suggesting that RG-1 arabinans are likely *in vivo* substrates for ARAF1.

### Phloem Fibres

One of the most conspicuous findings of the present study was the strong labelling of what appears to be the primary walls of the phloem fibres with LM5 (galactans), with the secondary walls being unlabelled. These fibres may be gelatinous fibres, which are sclerenchyma fibres that, in contrast to most sclerenchyma fibres, have an inner gelatinous or G-layer with a high content of axially oriented cellulose molecules and little or no heteroxylan or lignin (Harris, 2017). Such gelatinous fibres occur widely in plants, including the phloem (bast) fibres of stems and the tendrils of climbing plants (Gorshkova et al., 2018; Chery et al., 2021). As in the phloem fibres of chicory, the phloem (bast) fibres of flax stems contain (1→4)-β-galactans in their cell walls (Gorshkova et al., 2004; Gurjanov et al., 2008; Salnikov et al., 2008). However, immunomicroscopy with LM5 showed the labelling in the gelatinous phloem fibres of flax located in the secondary (gelatinous wall) adjacent to the plasma membrane (Andeme-Onzighi et al., 2000) or primary and secondary walls (His et al., 2001). We further found that the primary walls of chicory phloem fibres had small amounts of HG (both the JIM5 and the JIM7 epitope) and almost no (1→5)-α-arabinan (LM6 epitope). This provided a stark contrast to the phloem cap parenchyma cells with their intense labelling with JIM5 and LM6. The thick secondary walls of the phloem fibres were not labelled with any of the four monoclonal antibodies we used. This finding is consistent with the results obtained for the secondary walls of the gelatinous phloem fibres in the stems of hemp (*Cannabis sativa*; Blake et al., 2008) in which pectic polysaccharides were absent and cellulose was the only polysaccharide present. Further studies are needed on the chicory phloem fibres to determine if they are indeed gelatinous fibres. It is possible that the phloem fibres in the leaves we examined were immature non-gelatinous fibres in which the secondary walls had formed but not yet lignified.

## Data Availability Statement

The original contributions presented in the study are included in the article/supplementary material, further inquiries can be directed to the corresponding author/s.

## Author Contributions

XS, IA, PH, KJ, and SH conceived and planned the study. XS acquired funding and collected data. XS, IA, and PH analysed and interpreted data and prepared the tables, revised the manuscript. YH prepared the figures. XS and IA wrote the manuscript. All the authors approved the final version of the manuscript.

## Conflict of Interest

The authors declare that the research was conducted in the absence of any commercial or financial relationships that could be construed as a potential conflict of interest.

## Publisher’s Note

All claims expressed in this article are solely those of the authors and do not necessarily represent those of their affiliated organizations, or those of the publisher, the editors and the reviewers. Any product that may be evaluated in this article, or claim that may be made by its manufacturer, is not guaranteed or endorsed by the publisher.
